# Supplementary material for: Women’s Views on Multifactorial Breast Cancer Risk Assessment and Risk-Stratified Screening: A Population-Based Survey from Four Provinces in Canada
Source: J Pers Med. 2021 Feb 2;11(2):95. doi: 10.3390/jpm11020095 (PMC7912955; doi:10.3390/jpm11020095)
Supplement: Supplementary file 1 [file jpm-11-00095-s001.pdf]

Table S1. Summary of associations between participants' characteristics (excluding don't know and prefer not to answer) and outcomes variables from mutually-adjusted logistic regression models by statistical significance.

**OUTCOMES VARIABLES**	Using personal information* to assess BC risk? (A1)	Using genetic test results to assess BC risk? (A2)	Changing screening frequency depending of BC risk? (A3)	Providing personal information* to assess BC risk? (B1)	Providing sample of blood or saliva for genetic test to assess BC risk? (B2)	Having a mammogram to assess BC risk? (B3)	To have your BC risk level assessed? (C1)	To have your BC screening more often, if BC risk higher than average? (C2)	To have your BC screening less often, if BC average or lower than average? (C3)	Not to be offered any BC screening, if BC risk much lower than average? (C4)
**Positive response (%)**	**72.7**	**72.9**	**63.5**	**61.5**	**64.1**	**66.7**	**74.8**	**85.7**	**49.2**	**64.4**
Age groups	-	-	-	+	+	+	+	+	-	+
Province	-	-	-	-	++	-	-	-	-	-
Country of birth	-	-	-	-	-	-	-	-	-	-
Ethnicity	-	-	-	+	-	-	-	-	-	-
Education level	+	-	-	+	-	-	-	+	-	-
Marital status	-	-	+	+	+	-	-	-	-	-
Employment status	-	-	+	-	-	-	-	-	-	-
Total family income	+	+	-	+	+	++	-	++	+	-
Perceived health status	-	-	-	-	-	+	-	-	-	-
Ever had breast cancer	+	+	-	+	+	-	-	-	-	-
Perceived lifetime risk of breast cancer	++	++	+	+	++	+	++	++	++	++
Ever had a mammogram	+	+	-	+	+	++	++	++	-	++
Ever had a genetic test for breast cancer	-	-	-	-	-	+	-	-	-	+
Family history of genetic test for breast cancer	-	+	+	+	+	+	+	-	+	-

* Personal information like age, cancers in your family, having children, lifestyle factors, breast density and weight.

- = p-value > 0.05; + = p-value between 0.05 and 0.0001; ++ = p-value <0.0001.
